# A survey on detecting healthcare concept drift in AI/ML models from a finance perspective

**DOI:** 10.3389/frai.2022.955314

**Published:** 2023-04-17

**Authors:** Abdul Razak M. S., Nirmala C. R., Sreenivasa B. R., Husam Lahza, Hassan Fareed M. Lahza

**Affiliations:** ^1^Department of Computer Science and Engineering, Bapuji Institute of Engineering and Technology, Davangere, India; ^2^Department of Information Science and Engineering, Bapuji Institute of Engineering and Technology, Davangere, India; ^3^Department of Information Technology, Faculty of Computing and Information Technology, King Abdulaziz University, Jeddah, Saudi Arabia; ^4^Department of Information Systems, College of Computers and Information Systems, Umm Al-Qura University, Makkah, Saudi Arabia

**Keywords:** concept drift, data stream, drift detection methods, unsupervised learning, feature (interest) point selection

## Abstract

Data is incredibly significant in today's digital age because data represents facts and numbers from our regular life transactions. Data is no longer arriving in a static form; it is now arriving in a streaming fashion. Data streams are the arrival of limitless, continuous, and rapid data. The healthcare industry is a major generator of data streams. Processing data streams is extremely complex due to factors such as volume, pace, and variety. Data stream classification is difficult owing to idea drift. Concept drift occurs in supervised learning when the statistical properties of the target variable that the model predicts change unexpectedly. We focused on solving various forms of concept drift problems in healthcare data streams in this research, and we outlined the existing statistical and machine learning methodologies for dealing with concept drift. It also emphasizes the use of deep learning algorithms for concept drift detection and describes the various healthcare datasets utilized for concept drift detection in data stream categorization.

## Introduction

Machine Learning (ML) is a set of methods, techniques, and tools for diagnosing and prognosing medical issues (Kralj and Kuka, 1998). ML is used to forecast illness progression, extract medical knowledge for outcome study, plan and support therapy, and manage patients. ML is also used for data analysis, such as recognizing regularities in data by successfully dealing with defective data, interpreting continuous data used in the Intensive Care Unit, and intelligent alerts, which improves monitoring (Strausberg and Person, 1999). Successful machine learning approaches can help integrate computer-based systems into healthcare, making medical specialists' work easier and better, and enhancing efficiency and quality.

Society pays for healthcare services through healthcare finance. Healthcare finance includes accounting and financial management. Accounting measures a business's activities and finances in financial terms, while financial management (corporate finance) applies theory and concepts to help managers make better decisions.^1^ Depending on disease severity, many AI/ML models anticipate financial costs. Concept drift occurs as illness severity grows and treatment costs change. The model's alteration owing to data changes is called concept drift. In this study, we will discuss concept drift, its types, and strategies for handling concept drift in healthcare and financial data.

In our paper, we outline the following:

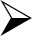
 Define concept drift in the healthcare domain and different drift types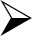 Review the work done for handling concept drift in the healthcare sector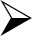 Classification techniques to handle concept drift

### Concept drift and its types

**Figure 1:**
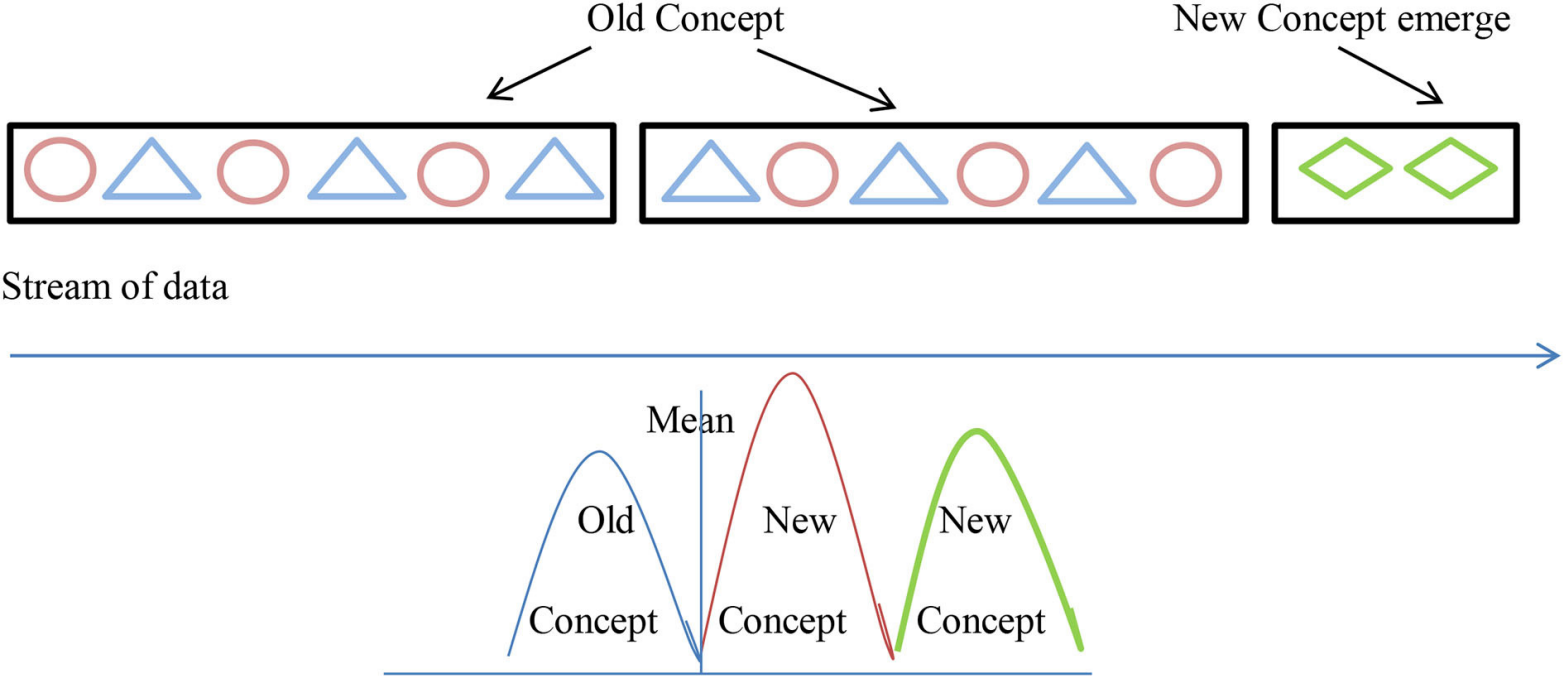
Concept drift.

Concept drift is most commonly associated with an online supervised learning scenario in which the relationship between the input data and the target variable changes over time (Gama et al., 2014). As a result of this, the error rate of the model increases which leads to the degradation of models' prediction results causing drift. Concept drift is also referred to as model drift or model decay in AI terminologies. Due to concept drift, the model misclassifies the data during the classification technique (Liu et al., 2017b) as shown in Figure 1.

Example: In the healthcare domain, the model created to predict the finance involved in treating a patient or insurance amount to be claimed from the company changes due to an increase in the complexity of the disease.

### Types of concept drift

**Figure 2:**
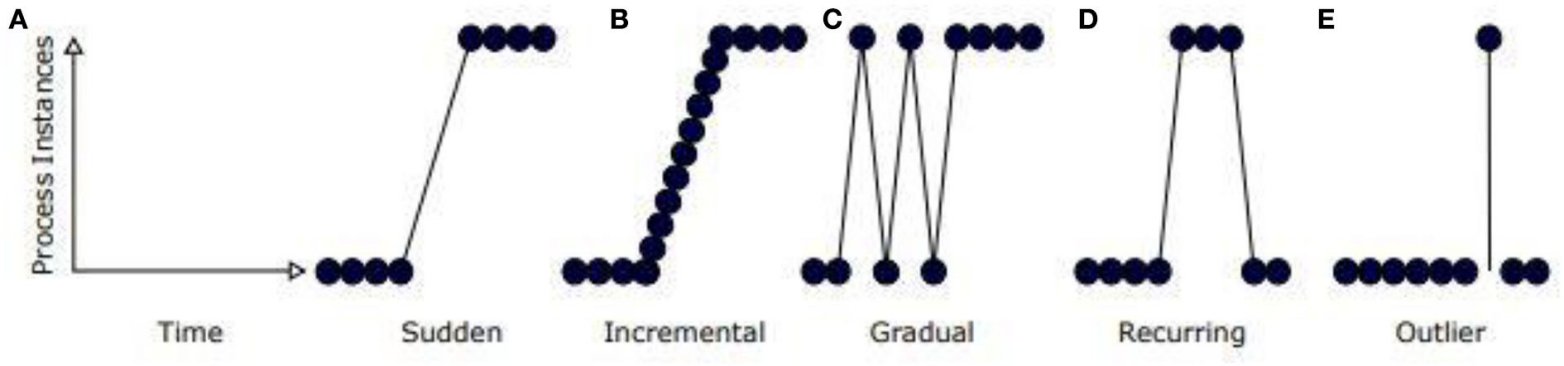
**(A–E)** Types of concept drift.

Figure 2 depicts different types of concept drift.

- **Sudden**—Sudden changes in health parameter values.
- **Gradual**—Concept is diminished slowly by another concept. For example, an increase in blood pressure leads to heart disease problems.
- **Recurring**—Changes reappear over the period. Example: Diabetic mellitus disease problems would reappear if there is a change in food habits.
- **Incremental**—Changes happen slowly over time.

Table 1 describes the different terminologies used to refer to different types of concept drift used in various works.

**Table 1:** Concept drift by the probabilistic source of change (Bayram et al., 2022).

Primary term	Sudden drift	Gradual drift	Recurring drift	Incremental drift
Alternative terms	Abrupt drift	Evolutionary drift	Recurring contexts	Stepwise drift
	Concept shift			
	Revolutionary drift		Replacing drift	Development drift
	Immediate drift			

**Figure 3:**
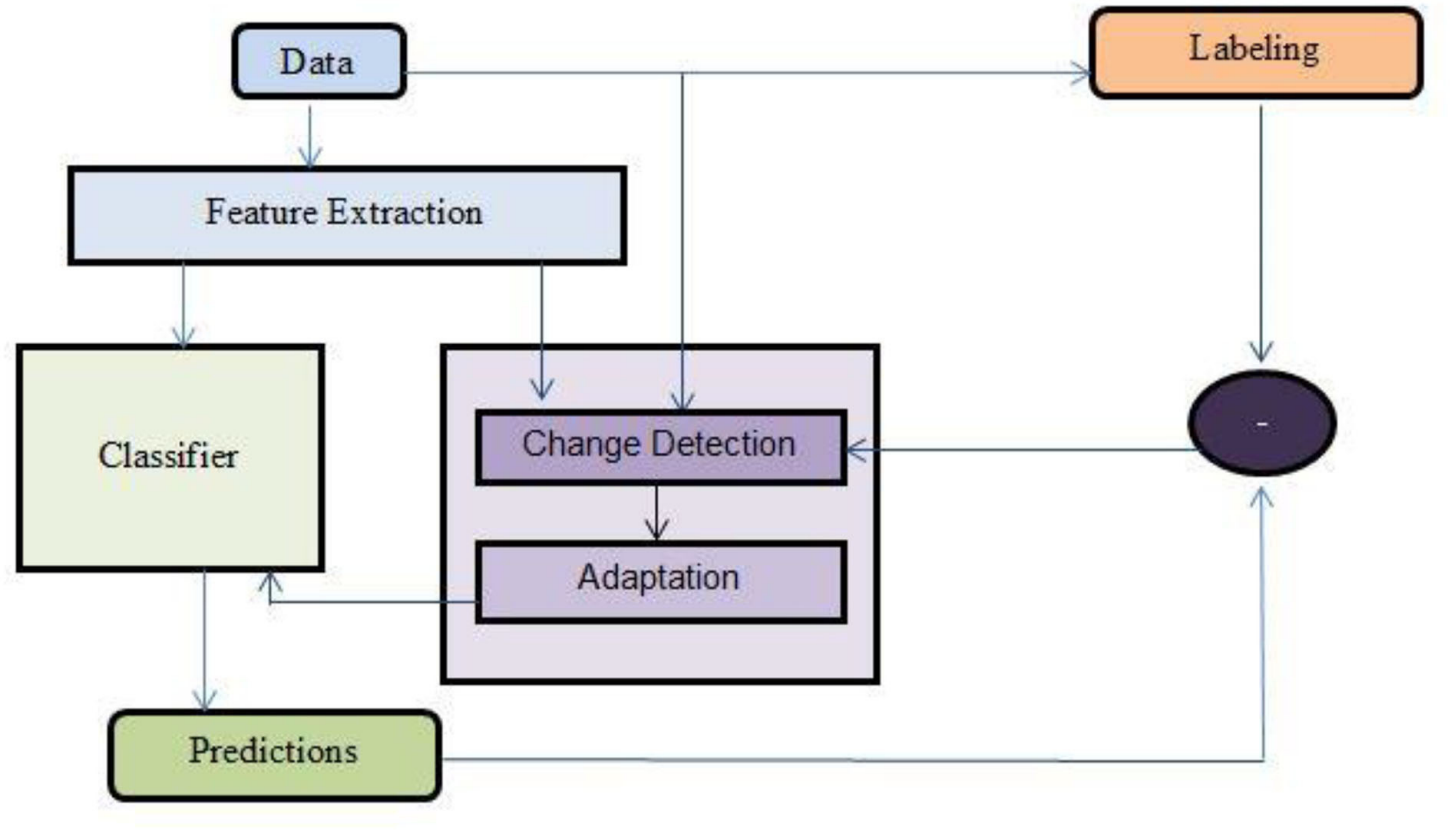
Different ways of occurrence of concept drift.

Figure 3 depicts the different ways of monitoring the occurrence of concept drift in data streams. The following are some of the ways:

- We can monitor the changes in data distribution
- We can monitor the feature changes in predicting the occurrence of concept drift.
- We can monitor the predictions of the classifier.
- We can monitor the labels of the data generated over time in finding the concept drift.

We picked potential papers based on the following inclusion criteria to determine applicable techniques.

- The method must be innovative for drift detection or integrate drift detectors into prediction systems.
- We listed all the papers related to different categories of drift detection from different authenticated journal databases.
- Papers related to concept drift in the healthcare sector are also listed.

**Figure 4:**
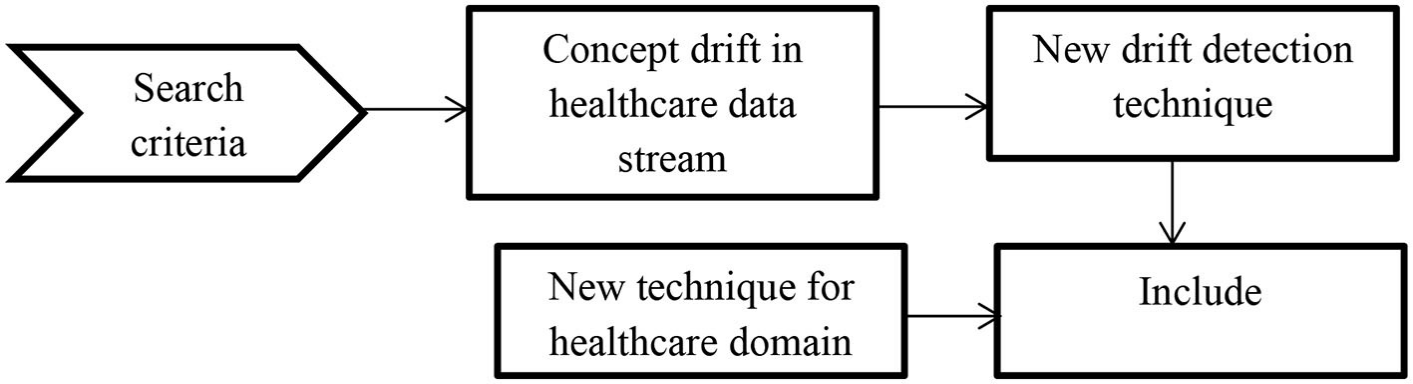
Systematic review process of concept drift.

In order to describe the review process involved in the manuscript to detect concept drift in healthcare application the following Figure 4 is used.

## Background review

### General framework to detect concept drift

**Figure 5:**
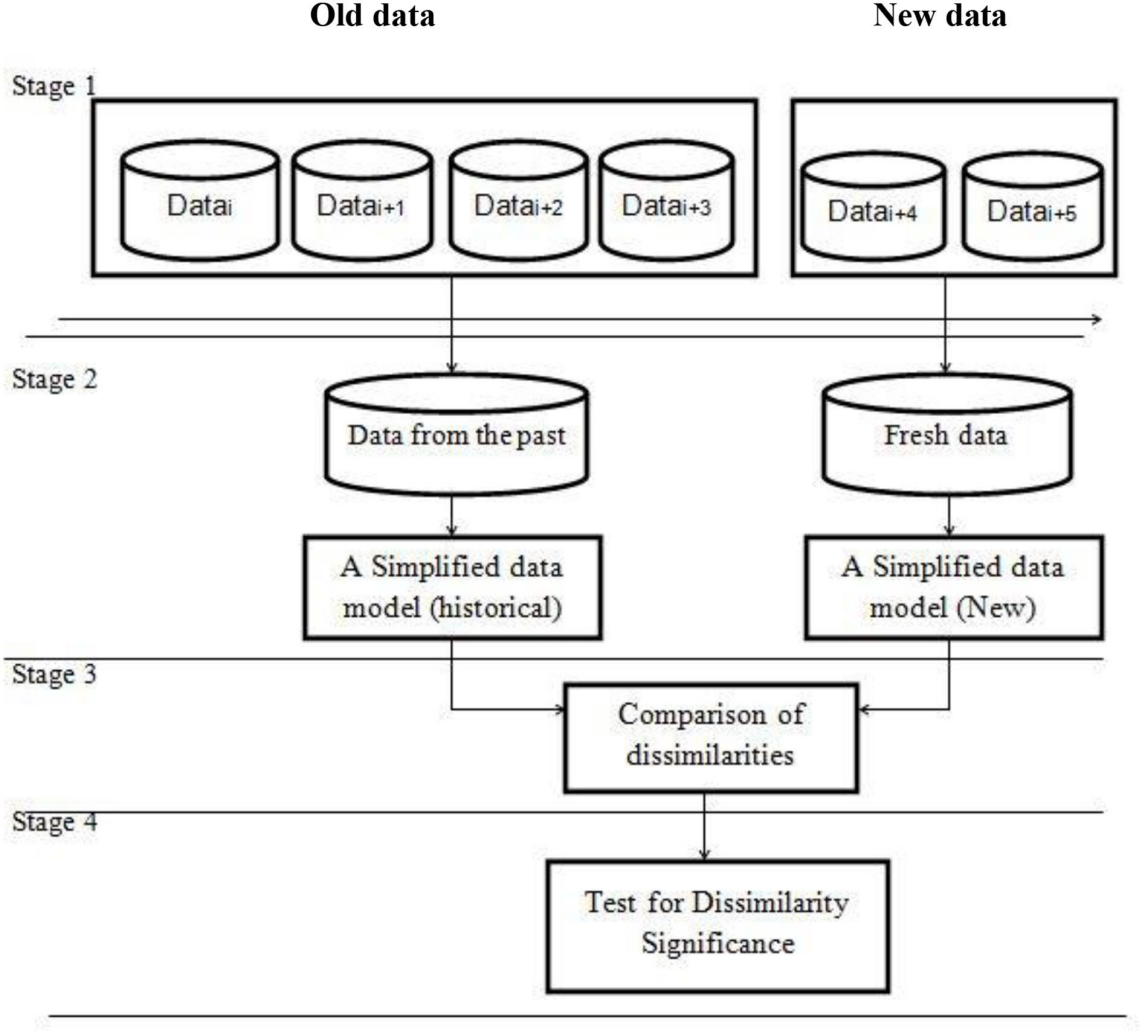
An overall framework for concept drift detection (Lu et al., 2019).

There are four stages in a generic framework for drift detection as shown in Figure 5.

***Stage 1 (Data Retrieval):*** Data retrieval extracts data chunks from data streams. Because a single data instance cannot infer the general distribution, data stream analysis jobs require knowledge of how to arrange data pieces into meaningful patterns (Lu et al., 2016; Ramirez-Gallego et al., 2017).
***Stage 2 (Data Modeling):*** Data modeling abstracts the returned data and extracts the sensitive features that most affect a system if they drift. Sample size reduction, or dimensionality reduction, to meet storage and online speed needs, is optional (Liu et al., 2017a).
***Stage 3 (Test Statistics Calculation):*** In Stage 3, distance or dissimilarity is estimated (Test Statistics Calculation). The drift's severity is assessed, and hypothesis test statistics are prepared. This is the hardest aspect of concept drift detection. How to define an accurate dissimilarity assessment is unknown. Dissimilarity measurements can evaluate clustering (Silva et al., 2013) and compare sample sets (Dries and Ruckert, 2009).
***Stage 4 (Hypothesis Test):*** Stage 4 (Hypothesis Test) uses the *p*-value to determine the statistical significance of Stage 3's change. Stage 3 test statistics are used to evaluate drift detection accuracy by showing their statistical bounds. Without Stage 4, Stage 3 test statistics cannot calculate the drift confidence interval, which reflects the likelihood that the change is due to concept drift rather than noise or random sample selection bias (Lu et al., 2014).

#### Concept drift in healthcare

On a multi-label hospital discharge dataset (Stiglic and Kokol, 2011) comprising diagnosis information, the suggested method employs relative risk and phi-correlation. The monthly discharge statistics and motion charts for visualization are used to detect concept drift. Static and dynamic ensemble classifiers are used to determine the accuracy and recommend the optimal classifier to use during concept drift.

Medical sensors that measure for general healthcare or rehabilitation (Toor et al., 2020) may be switched to ICU emergency procedures if necessary. When data have skewed class distributions, which is commonly the case with medical sensors' e-health data, detecting concept drifts becomes more difficult. The Reactive Drift Detection Method (RDDM) quickly finds long concepts. However, RDDM is error-prone and cannot handle class imbalance. The Enhanced Reactive Drift Detection Method (ERDDM) solves concept drift in class-imbalanced data streams.

We compared ERDDM to three recent techniques for prediction error, drift detection delay, latency, and data imbalance.

Clinicians triage patients referred to a medical facility using referral documents (Huggard et al., 2020), which contain free text and structured data. By training a model to predict triage decisions from referral documents, we may partially automate triage and make more efficient and methodical decisions. This task requires robustness against triage priority changes due to policy, budget, staffing, or other considerations. Concept drift occurs when document features and triage labels change. The model must be retrained to reflect these changes. This domain uses the unique calibrated drift detection method (CDDM). CDDM outperformed state-of-the-art detectors on benchmark and simulated medical triage datasets and had fewer feature drift-induced false positives.

Calibration drift detection alerts users to model performance degradation (Davis et al., 2020). Our detector maintains a rigorous calibration measure using dynamic calibration curves, a new method for tracking model performance as it grows. An adaptive windowing (Adwin) strategy monitors this calibration parameter for drift as data accumulate (Wang and Abraham, 2015).

In this study, a hip replacement dataset surgical prediction model was created (Davis et al., 2020). Concept drift is indicated by data distribution changes that increase mistake rate and classifier performance. The trigger-based ensemble method handles concept drift in surgical prediction by processing each sample and adapts the model quickly to data distribution changes.

Deep learning can detect early infection in CT, MRI, and X-Ray images of sick individuals from medical institutions or public databases. Analyzing infection rates and predicting outbreaks uses the same methodology. Many open-source pre-trained classification or segmentation models are available for the intended study. For example, transfer learning improves COVID-19 identification and prediction in medical picture datasets (Prashanth et al., 2022).

Table 2 describes the summary of concept drift detection methods in healthcare datasets. It briefs the drift detection method, the healthcare datasets used, the hypothesis test, features, and the given method's drawbacks.

**Table 2:** Drift detection methods in healthcare datasets.

**References**	**Drift detection methods**	**Health care datasets**	**Hypothesis test**	**Pros**	**Cons**
Stiglic and Kokol (2011)	Motion charts for drift detection in the detection of sudden concept drift	NHDS data	Relative risk and phi-correlation	Visualization helps select abnormally dynamic features	Detects only one type of drift. i.e., (sudden drift )
Toor et al. (2020)	Enhanced Reactive Drift Detection Method (ERDDM)	Medical Sensors measuring for general healthcare or rehabilitation.	–	To address the class imbalance, SMOTE was used.	Handles abrupt and gradual drifts.
Huggard et al. (2020)	calibrated drift detection method (CDDM)	benchmark and synthetic medical triage datasets	Nemenyi *post-hoc* test to compare the detection methods.	CDDM is h less prone to false positives.	A single system that can handle all changes in triage priorities.
Davis et al. (2020)	Adaptive windowing (Adwin)	Department of Veterans Affairs (VA)	–	Calibration curves show model performance over anticipated probability ranges. Loess smoothing or logistic regression is used to produce these curves. Addresses all types of drift	Accuracy in the detection of different types of drift can be improved.
Beyene et al. (2015)	Trigger-based Ensemble (TBE)	hip-replacement dataset	Nemenyi *post-hoc* test	Automate the prediction task of surgery.	The ensemble size does not become overly large

## Categories of concept drift detection

There are two categories of detecting concept drift:

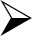
 Supervised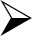 Unsupervised

### Supervised methods of concept drift detection categories

#### Performance-based Methods

This section examines performance-based concept drift detection techniques. Depending on the mechanism employed to identify performance dips, these techniques can be divided into one of several categories.

From the perspective of healthcare, performance-based techniques monitor the vital parameter values of patients' records. Any changes in the parameter values will alert the signal to take immediate actions. Following are some of the techniques used to monitor the performance of healthcare data:

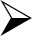
 Statistical process control/Error rate-based methods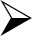 Window based methods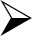 Sequential analysis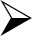 Ensemble methods

##### Statistical process control/error rate based methods

Statistical process control checks our model's error. This is especially crucial while running production as the performance changes over time. Thus, we would like to have a system that will issue an alarm if the model passes the specified error rate.

**Statistical process control methods are mentioned below:**

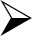
 ***DDM—Drift detection method***: It models a number of errors as a binomial random variable. The key is to monitor the error rate. The parameters to monitor the error rate includes μ that is average error rate and σ, i.e., standard deviation (Gama et al., 2004).

*p*_*t*_ = error rate of algorithm/probability of misclassification


(1) $$\begin{array}{l} \mu =np_{t}\text{ and }\sigma =\sqrt{\frac{p_{t}\left(1-p_{t}\right)}{n}} \end{array}$$

μ = mean, σ = Standarddeviation, *n* = numberofsamples

To alert if drift occurs, it uses the following equation


(2) $$\begin{array}{l} p_{t}+\sigma_{t}\geq p_{\min}+3\sigma_{\min} \end{array}$$

*p*_min_ = minimum value of the error rate

σ_min_= minimum value of standard deviation

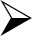
 ***EDDM—Early drift detection method:*** It uses the distance between two consecutive errors rather than a number of errors. The distance should stay constant and any variations in the distance lead to drift. The method is good at detecting gradual concept drift (Baena-Garcia et al., 2006).

Warn and start caching:


(3) $$\begin{array}{l} \frac{p_{t}+2\sigma_{t}}{p_{\max}+2\sigma_{\max}}\;<\;0.95 \end{array}$$

Alert and reset max


(4) $$\begin{array}{l} \frac{p_{t}+2\sigma_{t}}{p_{\max}+2\sigma_{\max}}<0.90 \end{array}$$

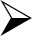
 **CUSUM Test:**

The CUSUM test (Manly and Mackenzie, 2000) calculates the difference of observed values from the mean and warns of concept drift if it exceeds a user-defined threshold. The cumulative total indicates concept drift when the error mean is significantly different from zero. The test alerts the user if the log-likelihood ratio of two probabilities before and after change exceeds this threshold.


(5) $$\begin{array}{l} T_{w}^{t}=log\frac{P\left(x_{w.}\ldots .,x_{t}|P2\right)}{P\left(x_{w,\ldots .,x_{t}}|P1\right)} \end{array}$$

The test declares a change when *g*_*t*_ is greater than the above equation threshold.


(6) $$\begin{array}{l} g_{t}\;=S_{t}-m_{t} \end{array}$$

$S_{t}=\overset{t}{\underset{i=1}{\sum }}s_{i}$ = ratio of log-likelihood between the two probabilities

$m_{t}=\;\underset{1\leq i\leq t}{\min}S_{i}\;$

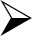
 **Page–Hinckley Test:**

The test detects an abrupt change of the average of a Gaussian signal and the detection process (Qahtan et al., 2015) consists of running two tests in parallel, testing between the no-change hypothesis H0: *r* > *n* and the change hypothesis H1: *r* > *n*.

To detect an increase in average, we calculate:


(7) $$\begin{array}{l} U_{n}=\;\sum_{i=1}^{n}\left(x_{i}-m_{0}-\frac{\delta_{m}}{2}\right)\;for\;\text{n}\geq 1\text{ and }U_{0}=\;0 \end{array}$$

$m_{n}=\underset{0\leq k\leq n}{\min}\left(U_{k}\right)$ for *n* ≥ 1

To detect a decrease in average, we calculate:


(8) $$\begin{array}{l} U_{n}=\sum_{i=1}^{n}\left(x_{i}-m_{0}-\frac{\delta_{m}}{2}\right)\;for\;\text{n}\geq 1\text{ and }U_{0}=0 \end{array}$$

$M_{n}=\max_{0\leq k\leq n}(T_{k})\text{for }n\geq \;1$

To alarm, we use *M*_*n*_−*T*_*n*_ ≥ τ

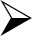
 **Hoeffding Drift Detection Method (HDDM):**

The Hoeffding Drift Detection Method (HDDM) (Frías-Blanco et al., 2015) enhances DDM by utilizing Hoeffding inequality to identify significant alterations in the performance estimate's moving average.

The Hoeffding bound is defined as:


(9) $$\begin{array}{l} \epsilon =\;\frac{R^{2}\ln\left(\frac{1}{\delta }\right)}{2n} \end{array}$$

ϵ = Hoeffding bound

R: Probability range. For probability, the range is 1, and for information gain, log c, where c is the number of classes.

δ: Confidence. 1 minus the required chance of choosing the right attribute at every given node.

n: Count of samples.

**The variants in the HDDM family include:**

✓ **Hoeffding drift detection with an A_test (HDDM**_**A**_**):** Methods for learning in data stream situations exist that compute confidence intervals for various parameters (such as error rate) while taking into account well-known distributions. The Hoeffding drift detection method with an A_test (Frías-Blanco et al., 2015) considers the difference between moving averages. It estimates the error ϵ_α_ given a significant level of at most α.
✓ **Hoeffding drift detection with weighted moving averages (HDDM**_**W**_**):** For weighted moving averages (Frías-Blanco et al., 2015), there is a broader statistical test that is quick and easy. Given that they are more likely to occur, the current real values are given greater weight than older ones in this situation.
✓ **Fast Hoeffding Drift Detection Method (FHDDM):** The FHDDM algorithm (Pesaranghader and Viktor, 2016) uses a sliding window and Hoeffding's inequality to compute and compare the highest probability of correct predictions with the most recent probability to detect drift.
✓ **Stacking fast Hoeffding drift detection method (FHDDMS):** The Stacking Hoeffding Drift Detection Approach (FHDDMS) (Pesaranghader et al., 2018), which maintains windows of various sizes, expands the FHDDM method. In other words, a short and a long sliding window are combined. This strategy's justification is to cut down on false negatives and detection delays. According to logic, a short window should be able to identify abrupt drifts more quickly than a lengthy window, which should do so with a lower rate of false negatives.
✓ **Additive FHDDMS (FHDDMS**_**add**_**):** FHDDMS_add_ detects abrupt concept drifts with shorter delays and reduces false negatives for gradual drifts.
✓ **Exponentially Weighted Moving Average (EWMA):** EWMA for Concept Drift Detection adapts EWMA charts to detect classifier error rate changes (Ross et al., 2012). Time t computes the EWMA estimator's dynamic standard deviation *Z*_*t*_ and error rate $p_{0,t}^{ˆ}$. Concept drift is indicated if

(10) $$\begin{array}{l} Z_{\text{t}}>p_{0,t}^{ˆ}+L\sigma_{\text{Zt}} \end{array}$$

The control limit, or parameter *L*, specifies how much Z_t_ must deviate from μ0 before a change is noted.

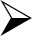
 **Extreme Learning Machine (ELM):** Extreme Learning Machine (ELM) (Huang et al., 2006) builds single-hidden layer feed-forward neural networks (SLFNs) by randomly selecting hidden nodes and calculating output weights. This technique provides good generalization performance at very fast learning speeds compared to gradient-based learning algorithms for feed-forward neural networks.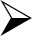 **Dynamic Extreme Machine Learning (DELM):** DELM (Xu and Wang, 2017) online learns a double hidden layer structure to improve ELM performance. ELM's notion drift alarm increases the classifier's generalization power by adding hidden layer nodes. If concept drift surpasses a threshold or ELM accuracy, the classifier will be withdrawn and retrained with new data to learn new concepts.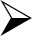 **Online sequential learning algorithm for feed-forward networks (OS-ELM):** Single-layer concealed a fast and accurate online sequential learning approach (OS-ELM) has built feed-forward neural networks with additive and radial basis functions (RBF) hidden nodes (Liang et al., 2006). Any limited non-constant piecewise continuous function can activate an additive node, and any integral piecewise continuous function can activate an RBF node. The algorithm can also process chunked data. Only the number of concealed nodes must be selected.

##### Sequential analysis-based methods

To determine how the context of the data stream has changed, the data instances are inspected one after the other. When the change in data distribution surpasses the predetermined threshold, it signals drift. The accuracy of the classifier is lowered as a result of concept drift. It can therefore be one of the methods used to identify concept drift in a particular data stream. Among the accuracy metrics of a classification model are recall, precision, F-measure, ROC, and AUC.

In Sequential analysis we monitor the contingency table. If the data are not stationary, we have different values in the table. If the data are not stationary, we have different values in the table. Rather than monitoring the contingency table values every time, we will monitor the four rates of the contingency table, i.e., precision, recall, sensitivity, and specificity to signal concept drift (Liu et al., 2016).

Liu et al. (2016) presented FP-ELM, which, like OS-ELM, can achieve incremental and on-line learning. Additionally, FP-ELM will apply a forgetting parameter to past training data based on current performance in order to adjust to possible changes after a new chunk is introduced.

Table 3 shows a categorical contingency table. A contingency table illustrates frequencies for specific combinations of two discrete random variables *X* and *Y*. The table cells contain mutually exclusive *X*–*Y* values.

**Table 3:** Contingency table (Agrahari and Singh, 2021).

	Actual →		
		0	1
Predicted ↓	0	TN	FN
	1	FP	TP

TP = True Positive FP = False Positive TN = True Negative FN = False Negative

Precision = $\frac{TP}{TP+FP}$, Recall = $\frac{TP}{TP+FN}$, sensitivity = $\frac{TN}{TN+FN}$, specificity = $\frac{TN}{TN+FP}$

This method sequentially evaluates prediction results as they become available and concept drift is flagged when a pre-defined threshold is met.

##### Window-based methods

**Figure 6:**
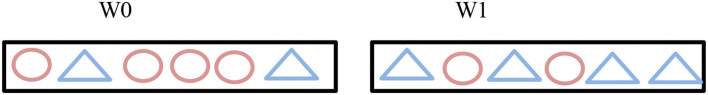
Window-based concept drift detection.

This method groups incoming data into a batch (or a window). Window-based methods have two windows. Figure 6 shows old data stream instances in the first window and new ones added afterward. These two window cases showed the drift and explained the data distribution change. This method can use either fixed or adjustable window sizes. A fixed window stays the same size during analysis. However, drift conditions change the adaptive window size. Drift shrinks the data window; no drift widens it (Agrahari and Singh, 2021).

From a healthcare perspective, the window can be considered as recording the patient's details every day or every hour and monitoring the changes over that period of time. Any improvements or fluctuations in that period will be carefully noticed and actions will be further taken.

The different window-based methods are as follows:

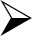
 **Adaptive windowing (ADWIN and ADWIN2):** Adaptive windowing (Bifet and Gavalda, 2007) considers all partitions of the window and compares the distribution between two windows. Any changes in distribution between two windows signals concept drift. For each partition, the method calculates the mean error rate and compares its absolute difference to a threshold based on the Hoeffding bound and if the subpartition is violated then it drops the last element in the window.

(11) $$\begin{array}{l} \text{Drop the last element}if\;|\mu_{0}-\mu_{1}|>\theta_{Hoeffding} \end{array}$$

Due to its low false positive and false negative rate, ADWIN performs effectively. The one-dimensional data that ADWIN can handle is its only drawback. It keeps different windows open. ADWIN2 is a modified version that uses less time and memory than ADWIN. The average distribution difference between two successive windows must be greater than a predefined threshold in order for drift to be detected. By identifying the slow, gradual drift, ADWIN2 gets around ADWIN's drawback. It requires O(log WS) memory and time if the window size is WS (Agrahari and Singh, 2021).

μ_0_ – Error rate of W0

μ_1_ – Error rate of W1

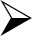
 **Detection Method Using Statistical Testing (STEPD):** Current and general accuracy is the rule. Two assumptions are made for this method: first, if the target concept is stationary, a classifier's accuracy for the most recent W examples will be equal to the overall accuracy from the start of learning; and second, a significantly lower recent accuracy signals that the concept is changing. Methods with an online classifier and monitoring its prediction errors during learning have been developed to detect concept drift in a limited number of samples. Nishida and Yamauchi (2007) created a detection approach that employs a statistical test of equal proportions.

The equation below calculates the statistic:


(12) $$T(r_{0},r_{r},n_{0},n_{r\;})=\;\frac{\left|\frac{r_{0}}{n_{0\;}}-\frac{r_{r}}{n_{r}}\right|-0.5(\frac{1}{n_{0}}+\frac{1}{n_{r}})}{\sqrt{\hat{p}(1-\hat{p}})\sqrt{\left(1/n_{0}\;+1/n_{r}\;\right)}}$$

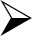
 **Wilcoxon Rank Sum Test Drift Detector (WSTD):** A novel two-window approach to STEPD-like concept drift detection in data streams. WSTD (de Barros et al., 2018) limits the older window size and employs the Wilcoxon rank sum statistical test instead of STEPD's test of equal proportions. The WSTD test equation is as follows:

(13) $$\begin{array}{l} z=\;\frac{\left(R-\mu_{R}\right)}{\sigma_{R}} \end{array}$$

μ_*R*_ = *population mean* = *n*1 × (*n*1+*n*2+1)

$\sigma_{R\;}=standar\;deiation=\;\sqrt{\frac{n1*n2*\left(n1+n2+1\right)}{12}}\;$

n1 = n2 = Size of the smallest and largest samples.

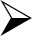
 **Fishers Exact Test:** The test proposed three approaches: Fisher Test Drift Detector (FTDD), Fisher Square Drift Detector (FSDD), and Fisher Proportions Drift Detector (FPDD) (de Lima Cabral and de Barros, 2018). Fisher's exact test was employed to get the *p*-value, which is the only distinction between these approaches and STEPD.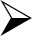 **Cosine Similarity Drift Detection (CSDD): The** Cosine Similarity Drift Detector (CSDD) produces the confusion matrix using a Positive Predictive Value (PPV) and the False Discovery (FDR) rates instead of TP and FP for each window. The Cosine Similarity (Hidalgo et al., 2019) between the vectors produced from the confusion matrices of the two windows indicates drift or warning.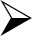 **McDiarmid Drift Detection Method (MDDM):** MDDM identifies concept drift using McDiarmid's inequality (Pesaranghader et al., 2017). MDDM works by sliding a window over the predictions and weighting the window entries. Recent entries are weighted more to highlight their importance. As examples are processed, the detection method compares the maximum weighted mean to the sliding window's weighted mean. When the weighted means diverge beyond the McDiarmid inequality, concept drift is inferred.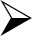 **Margin Density Drift Detection (MD3):** To signal drift, MD3 uses the number of samples mapped to a classifier's uncertainty zone (Sethi and Kantardzic, 2015).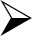 **Kolmogorov–Smirnov test (KS test):** A concept change detection technique called KSWIN (Kolmogorov–Smirnov Windowing) is based on the Kolmogorov–Smirnov (KS) statistical test. The KS test is a statistical test that makes no assumptions about the distribution of the underlying data. Data or performance distributions can be watched by KSWIN. The detector will accept an array of one-dimensional input. The KS test is run on identically sized windows, R and W. The distance of the empirical cumulative data distribution is compared using the KS test. KSWIN can identify concept drift if:

(14) $$\begin{array}{l} dist\left(R,W\right)>\;\sqrt{-ln\frac{\alpha }{r}} \end{array}$$

Because *R* and *W* are derived from the same distribution, if the difference in empirical data distributions between them is too great, concept drift can be identified

Raza et al. (2015) provide unique covariate shift-detection techniques based on a two-stage structure for both univariate and multivariate time-series. The first stage detects the covariate shift-point in non-stationary time-series using an exponentially weighted moving average (EWMA) model-based control chart in online mode. The second step confirms the first stage's shift detection using the Kolmogorov-Smirnov statistical hypothesis test (K-S test) for univariate time-series and the Hotelling T-Squared multivariate statistical hypothesis test for multivariate time-series.

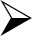
 **CIDD-ADODNN Deep learning framework:** The CIDD-ADODNN (Priya and Uthra, 2021) model classifies extremely imbalanced streaming data efficiently. The recommended adaptive synthetic (ADASYN) methodology weighs minority class examples based on learning difficulties to handle class imbalance data. Concept drift is detected using an adaptive sliding window (ADWIN).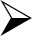 **Concept drift adaptation using Recurrent Neural Networks:** Recurrent neural networks (RNNs) are utilized to detect time series anomalies (Saurav et al., 2018). Since new data are added gradually, the model can adapt to data distribution changes. RNN predictions of the time series are used to discover anomalies and change points. A significant prediction error indicates deviant behavior.

##### Ensemble methods

**Figure 7:**
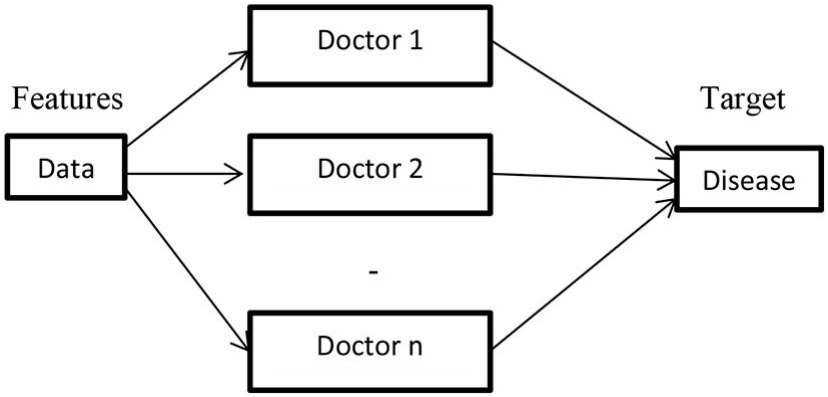
Ensemble methods.

Figure 7 depicts the ensemble method architecture. The above architecture has *n* models and combines the predictions of all models to predict the output. From a healthcare perspective, the ensemble method resembles the consulting decisions of multiple doctors to ensure the disease type and level before diagnosing.

The ensemble methods used for concept drift detection are as follows:

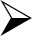
 **Streaming Ensemble Algorithm (SEA):** By adding a new learner for each new chunk of data until the maximum number of learners is reached, SEA (Street and Kim, 2001) automatically manages drift. Based on their performance with predictions, the learners are improved.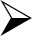 **Accuracy-Weighted Ensemble (AWE):** An Accuracy-Weighted Ensemble (Wang et al., 2003) is a group of classification models where each model is carefully weighted according to how accurately they are projected to classify the test data in a time-evolving environment. The ensemble ensures that it is effective and resilient to concept-drifting streams.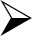 **Accuracy Updated Ensemble (AUE):** By utilizing online component classifiers and updating them in accordance with the current distribution, Accuracy Updated Ensemble (AUE) (Brzeziński and Stefanowski, 2011), a development of AWE, increases accuracy. Additional weighting function adjustments address issues with unintended classifier discarding seen in AWE.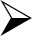 **Dynamic Weighted Majority (DWM):** Four strategies are employed by the dynamic weighted majority (DWM) (Kolter and Maloof, 2007) to counteract concept drift. It develops the ensemble's online learners, weights them depending on their performance, deposes them based on their performance, and adds fresh experts based on the ensemble's overall performance.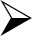 **Learn++.NSE**: A group of learners is trained in Learn++.NSE (Polikar et al., 2001) using examples of data chunks. The weighting of the training instances is based on the ensemble error for this particular case. Learn++.NSE increases the example's weight to 1 if the ensemble properly classifies it, otherwise, it is penalized to w_i_ = 1/e. Based on their mistakes in the previous and current chunks, the ensemble of learners is weighted using the sigmoid function.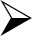 **Adaptive Random Forest (ARF):** ARF (Gomes et al., 2017) uses efficient resampling and adaptive operators to tackle concept drifts without data set optimization.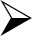 **DDD:** DDD regulates learner diversity by including low and high-diversity ensembles. The low diversity ensemble and high diversity ensemble are used after drift detection.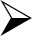 **DDE:** Bruno Maciel et al., 2015 made a small ensemble to control how three drift detectors work and block their signals at both the warning level and the drift level. Depending on how sensitive the DDE is, it needs a certain number of detectors to confirm an alarm or drift level. Another parameter is the type of drift mechanism that is used. But each sensitivity setting has a default detector setup that goes with it.

##### Data distribution methods

**Figure 8:**
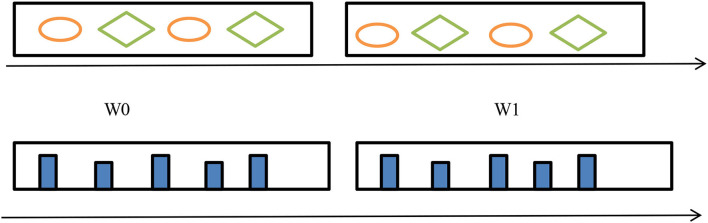
Feature distribution monitoring.

To determine the contextual shift, this kind of drift detection approach compares examples of recent and previous data. These techniques often examine the statistical significance and are used in conjunction with the window-based approach. The location of the drift can be determined by computing the change in data distribution. As a result, computational costs might result and it depicted in Figure 8.

From a healthcare perspective, the distribution of current vital health parameters are studied with the previous day/week or previous hour, and even before diagnosis and after diagnosis. The patient's health parameters are carefully observed and studied and, depending on the health parameter value distributions, the doctor can further extend the medications to the patient or discharge the patient from the hospital.

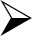
 SyncStream (Shao et al., 2014) is a prototype-based categorization model for evolving data streams that dynamically models time-changing ideas and offers local predictions. By constantly keeping a collection of prototypes in a new data structure known as the P-tree, SyncStream captures developing notions instead of learning a single model on a sliding window or ensemble learning. The prototypes are created using limited clustering and error-driven representativeness learning. Heuristics based on PCA and statistics are used to detect abrupt idea drift in data streams.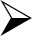 PCA-Based Change Detection Framework (Qahtan et al., 2015) methodology is built on estimating data for a subset of key constituents. Densities in reference and test windows are estimated and compared for each projection. Then, one of the divergence measures determines the change-score value. The largest change score among the several principal components is taken into account as the final change score by giving equal weight to all of the selected principal components.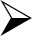 A technique by Ditzler and Polikar (2011) uses an adjustable threshold to calculate the Hellinger distance as a measure to determine whether drift exists between two batches of training data.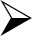 A brand-new test, outlined in Bu et al. (2018), for detecting changes without using the probability density function that works online with multidimensional inputs has been created and is based on the least squares density-difference estimation method. By using a reservoir sampling mechanism, the test can start running right away after configuration and does not require any assumptions about the distribution of the underlying data. Once the application designer has established a false positive rate, the requested thresholds to detect a change are automatically derived.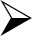 A local drift degree (LDD) (Liu et al., 2017a) measurement can track alterations in local density over time. After a drift, we synchronize the regional density disparities in accordance with LDD rather than suspending all historical data.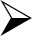 Reactive Robust Soft Learning Vector Quantization (RRSLVQ) (Raab et al., 2020) is a method for detecting concept drift that combines the Kolmogorov–Smirnov (KS) test with the Robust Soft Learning Vector Quantization (RSLVQ).

#### Multiple hypothesis based methods

These algorithms are unusual in that they employ several hypothesis tests to track concept drift in various ways. The two types of multiple-hypothesis tests are parallel and hierarchical (Lu et al., 2019).

From a healthcare perspective, before diagnosing any disease, multiple tests should be done mandatorily. For a pandemic disease like SARS-CoV-2, the virus that causes COVID-19, testing specimens from your nose or mouth, NAATs, such as PCR-based tests, are most often performed in a laboratory. Furthermore, antigen tests and MRI scans of the chest have to be done to know the severity of the disease.

Two-stage covariate shift identification tests are available for both univariate and multivariate time series. The first stage uses an exponentially weighted moving average (EWMA) control chart to locate the covariate shift point in a non-stationary time series online. The second stage validates the shift found in the previous stage with the Kolmogorov–Smirnov statistical hypothesis test (K–S test) for univariate time series and Hotelling's T-squared multivariate test.

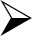
 The study by Yu et al. (2019) proposes a concept drift detection framework (LFR) for detecting concept drift and finding data points linked with the new concept. LFR can handle batch, stream, unbalanced, and user-specified parameters.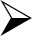 As proposed by Yu et al. (2018), a rapid concept drift detection ensemble (DDE) that integrates three concept drift detection algorithms to improve drift detections. Accuracy improves without affecting execution time.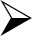 This article (Alippi and Roveri, 2008) proposes a pdf-free extension of the standard CUSUM using the CI-CUSUM test, which somehow inherits the extended CUSUM's detection skills and computational intelligence philosophy. Non-stationary data can be detected via the CI-CUSUM test.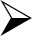 A novel hierarchical hypothesis testing framework with a Request-and-Reverify technique detects idea drifts by asking for labels only when needed. The unique paradigm offers hierarchical hypothesis testing with classification uncertainty (HHT-CU) and attribute-wise “goodness-of-fit” (HHT-AG).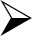 A hierarchical hypothesis testing (HHT) system that can detect and adjust to concept drift in unbalanced data labels (such as recurrent or irregular, gradual, or abrupt). HLFR, a new drift detector, is implemented using the HHT framework by switching to adaptive training.

### Unsupervised methods of concept drift detection categories are as follows

#### Clustering/novelty detection

**Figure 9:**
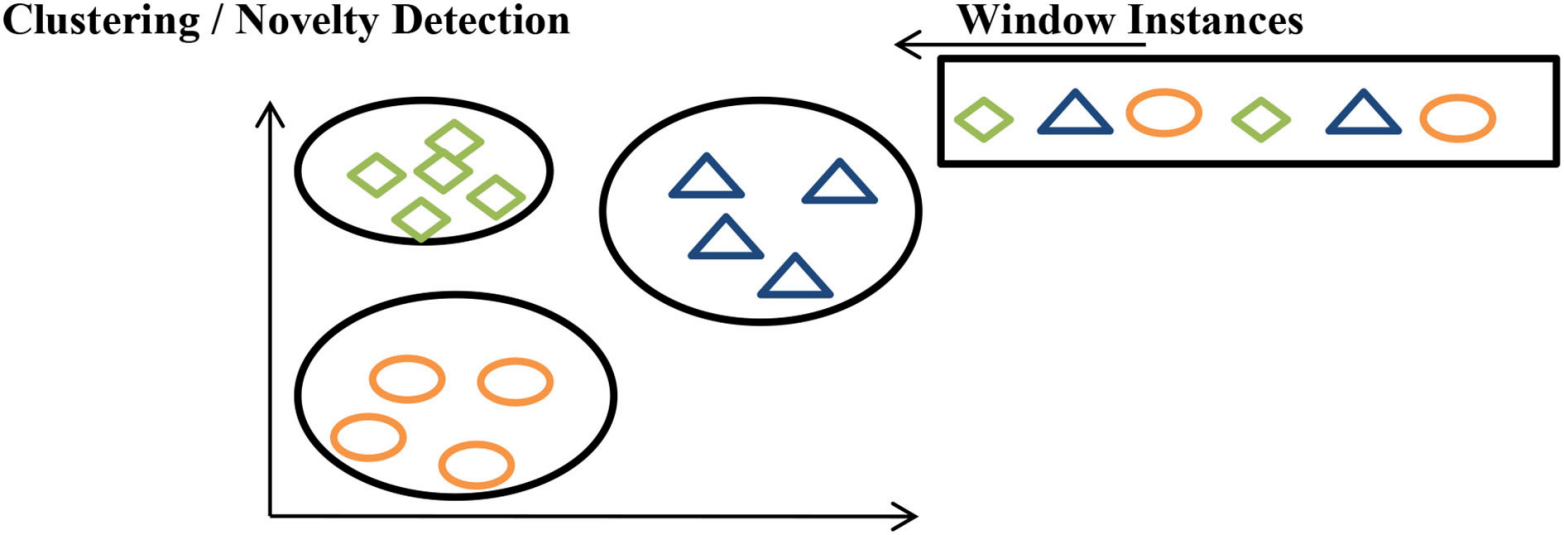
Clustering data streams.

In clustering, each batch of data is assigned to a particular group and if it is not assigned to any particular group, concept drift is declared, as shown in Figure 9.

From a healthcare perspective, we monitor all the health parameter values. After studying all health parameter values and reports the disease label is named.

The different clustering methods for stream data are as follows:

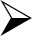
 **OLINDDA**—This method uses *k* means clustering and periodically merges known and unknown batches of data. If the latter, concept drift is flagged (Spinosa et al., 2007).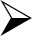 **MINAS**—This method uses micro clustering and to gain efficiency it uses incremental clustering (Faria et al., 2013).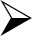 **DETECTNOD**—This method uses discrete cosine transform to estimate the distances efficiently (Hayat and Hashemi, 2010).

- **Woo-Ensemble**—This method treats outliers as potential emerging class centroids (Ryu et al., 2012).
- **ECSMiner**—Stores and uses cluster summary efficiently (Masud et al., 2011).
- **GC3**—This method uses grid-based clustering (Sethi et al., 2016).

#### Feature distribution monitoring

The idea is to monitor each feature individually. We monitor two sub-windows, W0 and W1, and compare their feature distribution either through Pearson correlation (Change of concept) or through Hellinger distance (HDDDM) (Lee and Magoules, 2012) as shown in Figure 9. If we have many features then the monitoring of features will be very difficult, so we can use principal component analysis (PCA) (Qahtan et al., 2015) to reduce features so that the monitoring of features will be easier.

#### Model-dependent monitoring

Unsupervised methods suffer from a high rate of false alarms because they constitute that any changes in observations are a reason for performance degradation. It is known that not all changes in observations lead to performance degradation. To reduce false alarm rates, we estimate the posterior probability (Dries and Ruckert, 2009).

The unsupervised methods under model-dependent monitoring are as follows:

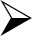
 **A—Distance method:** This method uses a generalized Kolmogorov–Smirnov distance to estimate the posterior probability.

## Healthcare datasets

Some of the various healthcare datasets used in concept drift detection include:

- **National Hospital Discharge Survey (NHDS) data**^2^ The dataset contains hospital discharge records for approximately 1% of US hospitals.
- **MIMIC-III** (Johnson et al., 2016)—a freely accessible critical care database: MIMIC-III (“Medical Information Mart for Intensive Care”) is a large, single-center database comprising information relating to patients admitted to critical care units at a large tertiary care hospital. Data include vital signs, medications, laboratory measurements, observations and notes charted by care providers, fluid balance, procedure codes, diagnostic codes, imaging reports, hospital length of stay, survival data, and more.
- **The Veterans Health Administration (VHA)** (Davis et al., 2020): It is one of three administrations within the Department of Veterans Affairs (VA), and is the largest integrated health system in the United States.
- **Hip-replacement dataset** (Clarke et al., 2015) from the orthopedics department of Blekinge hospital.
- **Il Paese Ritrovato Dataset**^3^ a healthcare facility located in Monza that was created for the residential care of people affected by Alzheimer's disease.

## Future research prospects

The following are some directions we can explore in the future:

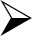
 **Drug Manufacturing Process:** Changes in drugs can impact the economy of the company. Early information about the drugs could stop or increase further production.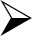 **Monitoring Health parameters during surgery:** Early information about the health parameters during surgery could help during diagnosis.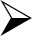 **Pandemic disease information:** COVID-19 has disturbed regular processes in the health sector. Prior information could help to avoid further problems.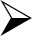 **Deep Learning Techniques to address concept drift problems**. New techniques to handle concept drift problems in the health industry could resolve many problems.

## Conclusion

This article investigated the concept of drift-handling algorithms designed for healthcare applications. It addresses supervised learning tasks where the aim is to generate a map of the feature instances, the target variables, and unsupervised learning tasks where there is an absence of class labels in the given instances. This article addresses the machine learning algorithms used to handle concept drift for medical domain problems. It also addresses different types of concept drift and how to handle them using implicit and explicit approaches. Different techniques for handling concept drift in the medical sector can be incorporated as future scope.

## Data availability statement

The original contributions presented in the study are included in the article/supplementary material, further inquiries can be directed to the corresponding author/s.

## Author contributions

Conceptualization: AM, SB, and HL. Data curation, methodology, writing—original draft, and formal analysis: AM and NC. Funding acquisition: HL. Investigation: AM and SB. Supervision: NC and SB. Validation: HML, SB, and AM. Writing—review and editing: SB, HML, HL, and AM.

## Footnotes

^1^Available online at: https://account.ache.org/iweb/upload/ReiterSong_Ch1-4e0b591e.pdf.

^2^Available online at: https://www.cdc.gov/nchs/nhds/nhds_questionnaires.htm.

^3^Available online at: https://www.cfsitalia.com/en/projects/il-paese-ritrovato/.
